# Dim light at night disturbs the daily sleep-wake cycle in the rat

**DOI:** 10.1038/srep35662

**Published:** 2016-10-20

**Authors:** Dirk Jan Stenvers, Rick van Dorp, Ewout Foppen, Jorge Mendoza, Anne-Loes Opperhuizen, Eric Fliers, Peter H. Bisschop, Johanna H. Meijer, Andries Kalsbeek, Tom Deboer

**Affiliations:** 1 Department of Endocrinology and Metabolism, Academic Medical Center (AMC), University of Amsterdam, Amsterdam, The Netherlands; 2 Institute of Cellular and Integrative Neurosciences, CNRS UPR3212, University of Strasbourg, France; 3 Hypothalamic Integration Mechanisms, Netherlands Institute for Neuroscience (NIN), Royal Netherlands Academy of Arts and Sciences, Amsterdam, The Netherlands; 4 Laboratory for Neurophysiology, Department of Molecular Cell Biology, Leiden University Medical Center, Leiden, The Netherlands

## Abstract

Exposure to light at night (LAN) is associated with insomnia in humans. Light provides the main input to the master clock in the hypothalamic suprachiasmatic nucleus (SCN) that coordinates the sleep-wake cycle. We aimed to develop a rodent model for the effects of LAN on sleep. Therefore, we exposed male Wistar rats to either a 12 h light (150–200lux):12 h dark (LD) schedule or a 12 h light (150–200 lux):12 h dim white light (5 lux) (LDim) schedule. LDim acutely decreased the amplitude of daily rhythms of REM and NREM sleep, with a further decrease over the following days. LDim diminished the rhythms of 1) the circadian 16–19 Hz frequency domain within the NREM sleep EEG, and 2) SCN clock gene expression. LDim also induced internal desynchronization in locomotor activity by introducing a free running rhythm with a period of ~25 h next to the entrained 24 h rhythm. LDim did not affect body weight or glucose tolerance. In conclusion, we introduce the first rodent model for disturbed circadian control of sleep due to LAN. We show that internal desynchronization is possible in a 24 h L:D cycle which suggests that a similar desynchronization may explain the association between LAN and human insomnia.

Millions of humans and animals around the world are exposed to artificial light during the night, when the natural environment should be dark^1^,^2^. In mammals, environmental light is the main input that synchronizes the master circadian clock in the hypothalamic suprachiasmatic nucleus (SCN) to the outside world. The SCN communicates the external time of day to other brain areas, including areas that regulate sleep and food intake such as the ventrolateral preoptic nucleus and the lateral hypothalamus^3^. The SCN also communicates with peripheral clocks in metabolic tissues such as liver and adipose tissue^4^, and regulates glucose metabolism^5^. Sleep and metabolism are tightly interconnected, since sleep quality directly affects human glucose metabolism^6^ and people with metabolic syndrome showed disturbed sleep^7^. Thus, exposure to artificial light at night may contribute to the high prevalence of insomnia^8^ and type 2 diabetes^9^,^10^.

Indeed, observational studies in humans showed that exposure to bedroom light at night is associated with insomnia^11^, obesity^12^,^13^, and dyslipidemia^13^. In addition, in two small studies in healthy humans, exposure to dim light during sleep at night decreased sleep depth and increased arousal frequency^14^,^15^. In nocturnal rodents, bright light at night increased sleep^16^ and decreased food intake^17^ within minutes. Furthermore, long term exposure to constant light disrupted circadian rhythmic behavior^18^,^19^,^20^, decreased total time spent awake^20^ and caused obesity and type 2 diabetes^18^,^19^ in rodents. As constant light conditions are uncommon outside the laboratory, dim light at night is a better model of exposure to artificial light at night. One study in free-living birds showed that light pollution with 2 lux at night acutely decreased total sleep time^21^. In Sprague Dawley rats, dim light at night decreased the amplitude of hormonal rhythms^22^,^23^. Nelson’s laboratory extensively studied the effect of dim light at night in Swiss Webster mice, and their studies showed that 5 lux light at night reduced the amplitude of the daily rhythms of locomotor activity and food intake, and induced obesity and diabetes^18^,^24^, whereas dim light at night had no significant effects on sleep distribution^25^.

To our knowledge, at present there is no working rodent model to investigate the mechanism underlying the disturbing effects of dim light at night on sleep. The rat provides more opportunities for physiological measurements of sleep and metabolism, since the larger size of the animal facilitates higher volume plasma sampling, targeted hypothalamic manipulations, and electrophysiological studies as compared to the mouse^5^,^26^. Therefore, in the present study we investigated the effects of 5 lux dim light at night on sleep-wake behavior and energy metabolism in Wistar rats.

## Results

### Vigilance states and sleep architecture

**Figure 1:**
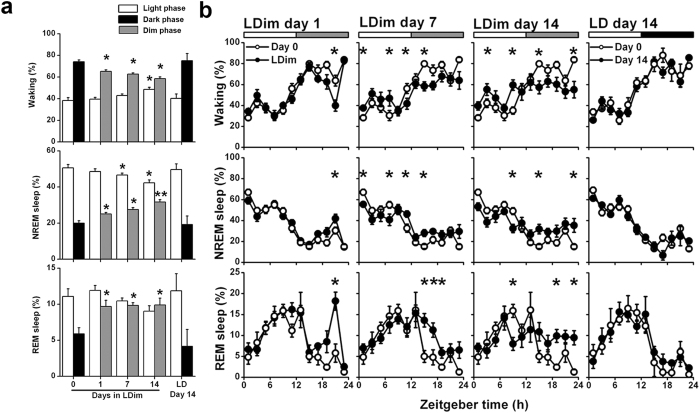
LDim reduces the daily rhythms in vigilance state distribution. Rats were exposed to LDim (n = 8) or LD (n = 5) and 24-h EEG/EMG recordings were performed on day 0 (baseline), day 1, day 7 and day 14. (**a**) LDim instantaneously decreased the amplitude of the daily rhythm in wakefulness (top), non-rapid eye movement (NREM) sleep (middle) and rapid eye movement (REM) sleep (bottom) with a further decrease over the following days. Control animals in LD showed no change in vigilance state distribution after 14 days. White bars: light phase, black bars: dark phase, grey bars: dim phase. (**b**) Percentage of time of 2-h intervals spent awake, in NREM sleep and in REM sleep. The baseline measurement at day 0 (open circles) is repeatedly plotted to facilitate comparison with subsequent days (closed circles). Registrations on day 0 and day 1 started at ZT12, recordings on day 7 and day 14 started at ZT0, but all data are plotted from ZT0 for convenience. Data are represented as mean ± SEM. If a significant main effect was detected, post hoc tests for differences from baseline were performed with student’s *t*-test and indicated by asterisks (P < 0.05) or double asterisks (P < 0.001).

To investigate the effects of light at night on sleep-wake behavior we exposed rats to either a 12 h light (150–200 lux):12 h dark (LD) schedule or a 12 h light (150–200 lux):12 h dim white light (5 lux) (LDim) schedule and performed EEG/EMG recordings. LDim strongly diminished the amplitude of the daily rhythm in all three vigilance states. Introduction of LDim decreased the time spent awake in the first dim phase compared to the baseline dark phase, with a further decrease over the following days. LDim gradually increased the time spent awake in the light phase (*Phase* P < 0.001, *Day* P = 0.136, *Interaction* P < 0.001; Fig. 1). Specifically, LDim reduced the duration of waking episodes during the dark (dim) phase and increased the duration of waking episodes during the light phase (Supplementary Figure 1). LDim also increased the time spent in NREM sleep in the first dim phase compared to the baseline dark phase, with a further increase over the days. LDim gradually decreased the time spent in NREM sleep in the light phase (*Phase* P < 0.001, *Day* P < 0.001, *Interaction* P < 0.001; Fig. 1). Specifically, LDim increased NREM sleep episode duration in the dark (dim) phase and reduced NREM sleep episode duration in the light phase (Supplementary Figure 1). Finally, LDim directly increased the time spent in REM sleep during the first dim phase compared to the baseline dark phase, and gradually decreased the time spent in REM sleep during the light phase (*Phase* P = 0.002, *Day* P = 0.003, *Interaction* P = 0.002; Fig. 1). The increase in REM sleep during the dark (dim) phase was mainly due to increased REM sleep episode numbers (Supplementary Figure 1). In LD, the daily rhythm in the vigilance states did not significantly change over the 14 days period (Fig. 1). Total 24 h sleep time was not significantly affected by LDim (LD: 43.8 ± 1.7%, 14 days LDim: 47.2 ± 1.0%, P = 0.293).

**Figure 2:**
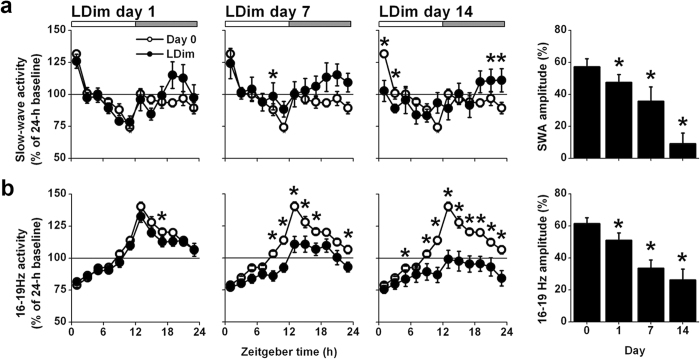
LDim reduces the daily rhythm of slow wave activity and 16–19 Hz activity within NREM sleep. (**a**) LDim (closed circles) decreased the amplitude of the daily rhythm in slow wave activity (SWA) compared to the baseline measurement (open circles). (**b**) LDim also strongly decreased the amplitude of 16–19 Hz activity compared to baseline. Data are expressed as percentage of the 24-h average at baseline. Registrations on day 0 and day 1 started at ZT12, but all data are plotted from ZT0 for convenience. Data are represented as mean ± SEM. If a significant main effect was detected, post hoc tests for differences from baseline were performed with student’s *t*-test and indicated by asterisks (P < 0.05) or double asterisks (P < 0.001).

In order to investigate homeostatic sleep drive, we analyzed the rhythm of slow wave activity (SWA, EEG power density between 0.75–4.0 Hz). SWA showed a clear day/night rhythm with a peak at the beginning and a trough at the end of the light phase. LDim gradually decreased the amplitude of the SWA rhythm (*Day* P = 0.001; Fig. 2a).

When analyzing the changes in power density of all frequencies between 0.25 and 25.0 Hz within the NREM sleep EEG, we observed reduced power in the 16–19 Hz frequency domain in the course of 14 days LDim (P < 0.05, after significant *Interaction* P < 0.05). Therefore we analyzed the rhythm of this frequency domain in more detail. The 16–19 Hz frequency domain showed a pronounced daily rhythm at baseline with a peak at the beginning of the dark phase. LDim strongly reduced the peak and the amplitude of this 16–19 Hz rhythm (*Day* P < 0.001; Fig. 2b).

### Circadian locomotor activity

**Figure 3:**
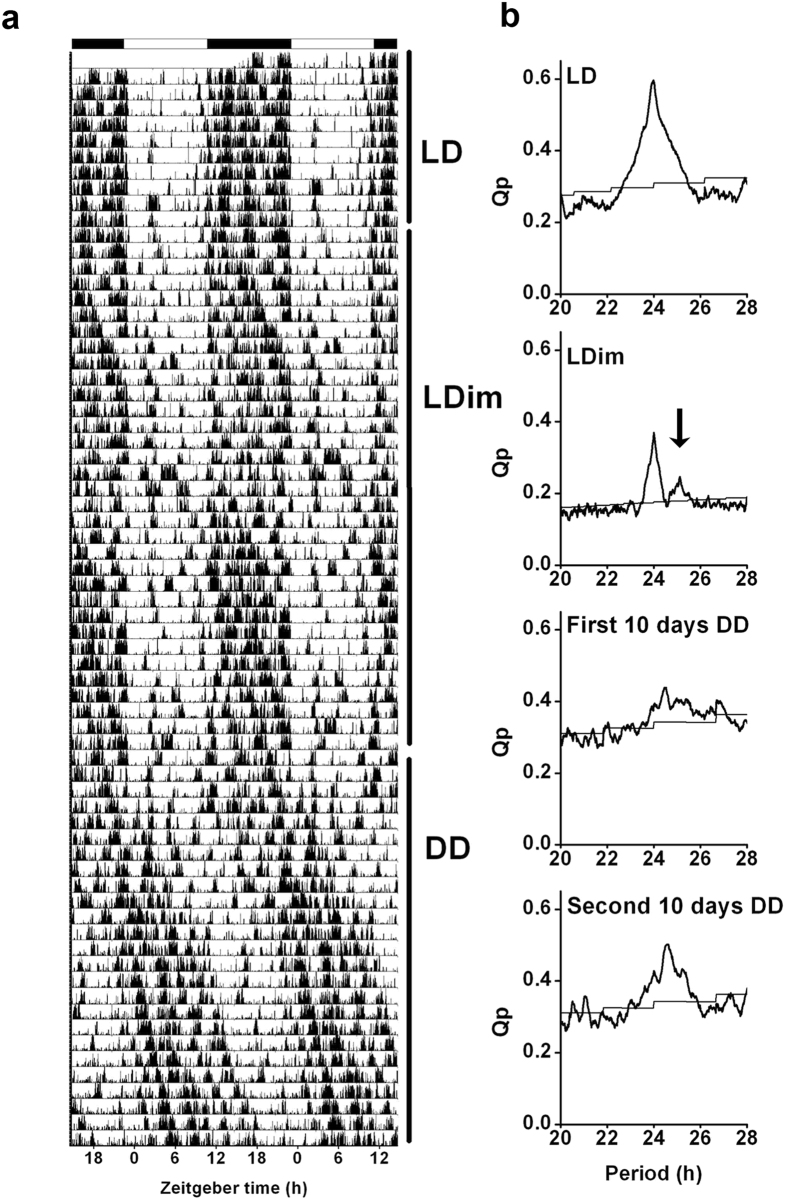
LDim induces an endogenous free run. Individual example of (**a**) a representative actogram and (**b**) the corresponding periodogram from circadian experiment A. The rat was first exposed to LD for 10 days, and subsequently to LDim for 30 days and constant darkness (DD) for 20 days. LDim exposure reduced the rhythmic strength of the 24 h rhythm and induced a second free running rhythm of 25.1 h (arrow) that interfered with the 24 h rhythm. During the first 10 days of DD, rhythmic strength was low, but during the following 10 days DD rhythmic strength returned.

To further investigate the effect of LDim exposure on the circadian timing system, we exposed male Wistar rats to 10 days LD followed by 30 days LDim and 20 days constant darkness (DD). Subsequently, the animals were re-entrained during 30 days to LD and finally the animals were released into DD for 10 days (circadian experiment A). LDim reduced the strength of the 24 h rhythm, and induced a second, interacting, free running rhythm with a period of 25.1 ± 0.0 h (see arrow in Fig. 3b). This interacting free running rhythm was observed in all animals exposed to LDim. After transfer from LDim to DD, rhythmic strength remained low in the first ten days with a period similar to the second rhythmic component in LDim, followed by an increase in rhythmic strength in the following 10 days (Fig. 3 and Table 1). Three out of 8 animals were virtually arrhythmic in the first days after transfer to DD (in these animals no significant period could be detected in the first 7 days after release in DD) and showed an improvement of the rhythm in the course of DD. After re-entrainment to LD and transfer from LD to DD all animals showed a free running period of 24.2 ± 0.1 h in DD.

As a control, we verified whether the occurrence of two interacting free running rhythms was specific for LDim and not due to continuous exposure to minimally 5 lux, by exposing Male Wistar rats consecutively to at least 10 days LD, 16 days constant dim light (DimDim), 22 days constant light (LL), 32 days DimDim, 14 days LD, and 16 days DimDim (circadian experiment B). DimDim exposure induced period lengthening to 24.9 ± 0.3 hr, but in contrast to LDim exposure, no dual rhythms were observed (Table 1, Supplementary Figure 2). LL exposure induced arrhythmic behavior and subsequent DimDim exposure did not restore rhythmicity. After re-entrainment to LD and re-exposure to DimDim, again period lengthening was observed.

### SCN clock gene expression

**Figure 4:**
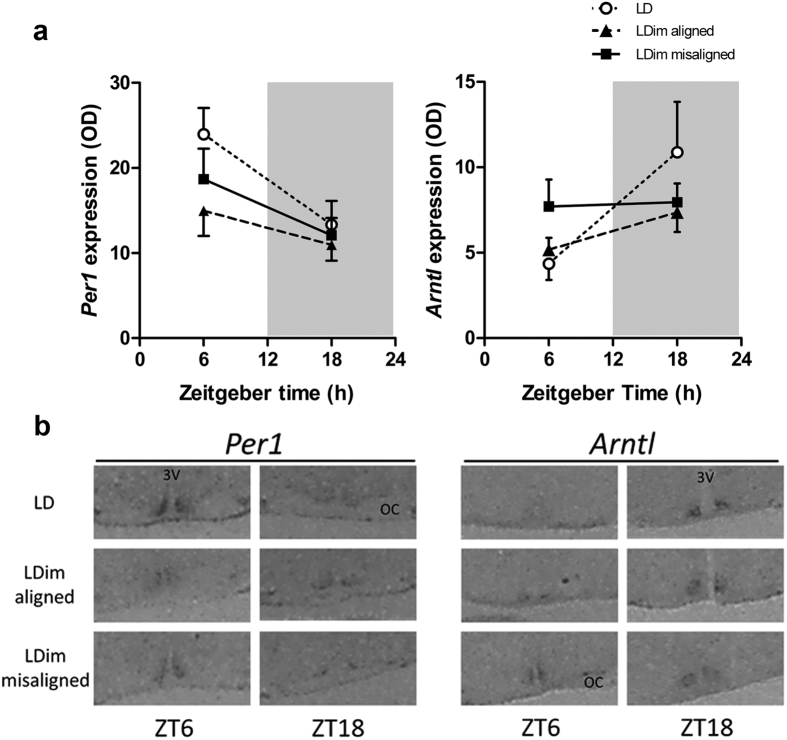
LDim reduces the rhythm of *Per1* and *Arntl* expression in the suprachiasmatic nucleus. Animals were exposed to LDim or LD and sacrificed at ZT6 or ZT18, LDim animals were sacrificed when the two rhythmic components in locomotor behavior were aligned or misaligned. (**a**) *In situ* hybridization detected a diurnal variation (P = 0.004) in *Per1* expression, and post hoc testing revealed that the effect of *Time* was only significant in the LD group (P = 0.025), but not in the LDim-aligned (P = 0.272) and LDim-misaligned (P = 0.136) groups. *Arntl* expression in the SCN also showed a significant diurnal variation (P = 0.027). Post hoc testing showed that the diurnal variation nearly reached significance in the LD group (P = 0.058) but not in the LDim-aligned (P = 0.130) and LDim-misaligned (P = 0.899) groups. (**b**) Representative *in situ* hybridizations. OD: Optical density. White circles: LD group. Black triangles: LDim aligned group. Black squares: LDim misaligned group. 3V: Third ventricle. OC: optic chiasm. Data are means ± SEM; n = 7 per group.

In order to investigate the representation within the SCN of the two rhythmic components observed in locomotor behavior, we analyzed SCN clock gene expression at the time when the two rhythmic components in behavior were aligned or misaligned. *Per1* expression in the SCN showed a significant diurnal variation with higher expression at ZT6 compared to ZT18 (*Time* P = 0.004, *Group* P = 0.137, *Interaction* P = 0.495). Post hoc tests showed that the effect of *Time* was only significant in the LD group (P = 0.025), but not in the LDim-aligned (P = 0.272) and LDim-misaligned (P = 0.136) groups (Fig. 4). There was no difference in dorsal/ventral ratio between ZT6 and ZT18 in misaligned animals (P = 0.324).

*Arntl* expression in the SCN showed a significant diurnal variation in antiphase to *Per1*, with lower expression at ZT6 compared to ZT18 (*Time* P = 0.027, *Group* P = 0.574, *Interaction* P = 0.145). Post hoc testing showed that the diurnal variation nearly reached significance in the LD group (P = 0.058), but not in the LDim-aligned (P = 0.130) and LDim-misaligned (P = 0.899) groups (Fig. 4). There was no difference in dorsal/ventral ratio between ZT6 and ZT18 in misaligned animals (P = 0.176).

### Energy metabolism and body weight

Previous experiments in mice showed that dim light at night can influence energy metabolism^18^. Therefore, we investigated the effects of LDim compared to LD on food intake, energy expenditure, and body weight, both on a chow diet and on a high fat diet (HFD), with metabolic cages. On a chow diet, LDim induced a decrease of the amplitude of the daily rhythm of food intake from 84 ± 2% dark phase food intake at baseline to 59 ± 2% dark phase food intake in week 7. This decrease was much stronger compared to the amplitude decrease in LD from 83 ± 2% dark phase food intake at baseline to 77 ± 3% dark phase food intake in week 7 (*Light schedule* P = 0.426, *Week* P < 0.001, *Interaction* P = 0.026). LDim also induced a decrease in the daily rhythm of energy expenditure compared to the LD animals (*Light schedule* P = 0.212, *Week* P = 0.001, *Interaction* P = 0.003) (Supplementary Figure 3). Animals exposed to LD showed a small increase over time of total 24-h food intake compared animals in LDim (*Light schedule* P = 0.927, *Week* P = 0.002, *Interaction* P = 0.025), but total 24-h energy expenditure did not differ between LDim and LD (*Light schedule* P = 0.908, *Week* P < 0.001, *Interaction* P = 0.084) (Supplementary Table 1).

**Figure 5:**
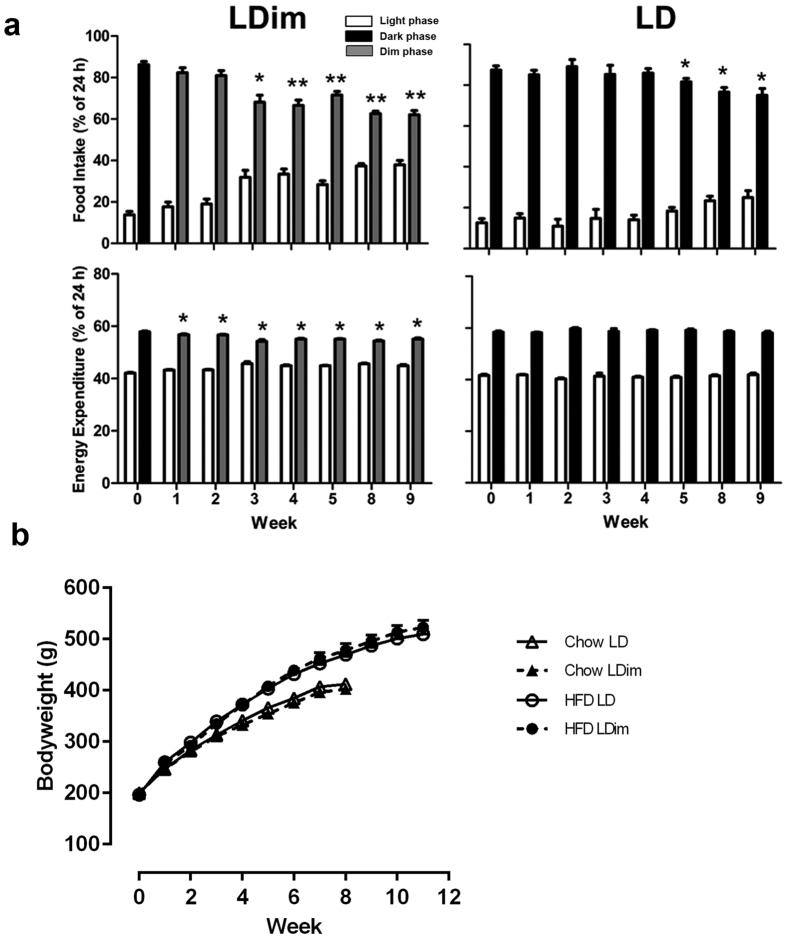
LDim reduces the daily rhythm in food intake and energy expenditure during a high fat diet. (**a**) Percentage of 24-h food intake and energy expenditure during the light (white bars), dark (black bars) and dim (grey bars) phase. LDim caused a stronger decrease of food intake and energy expenditure during the dim phase compared to the dark phase in LD. On a HFD, a number of 110 representative 48-h recordings were obtained out of 128 attempts (86%). Missing data were due to technical or scheduling issues. Statistical analyses were performed on dim/dark-phase percentages. Significant differences from baseline are indicated by asterisks (P < 0.05) or double asterisks (P < 0.001) Data are means ± SEM; n = 16. (**b**) LDim (open symbols) does not affect body weight increase either on a chow (triangles) or on a high fat diet (HFD, circles) compared to LD (closed symbols). Data are means ± SEM. Chow: n = 8; HFD: n = 16.

On a HFD, LDim again induced a stronger reduction of the daily rhythm of food intake compared to LD (*Light schedule* P = 0.132, *Week* P < 0.001, *Interaction* P = 0.001) (Fig. 5), however, total 24-h food intake was not significantly affected by LDim (*Light schedule* P = 0.079, *Week* P = 0.321, *Interaction* P = 0.238) (Supplementary Table 1). LDim gradually decreased the daily rhythm in energy expenditure compared to LD (*Light schedule* P = 0.002, *Week* P < 0.001, *Interaction* P < 0.001 (Supplementary Figure 3). Total 24-h energy expenditure was not affected by LDim (*Light schedule* P = 0.990, *Week* P < 0.001, *Interaction* P = 0.249) (Supplementary Table 1).

The increase in body weight did not differ between LDim and LD, neither on a chow diet nor on a HFD (Fig. 5 and Supplementary Table 2). White adipose tissue (WAT), adrenal, and thymus weights were not different between LDim and LD on either a chow diet or a HFD (Supplementary Table 2).

Glucose tolerance as assessed with the plasma glucose incremental area under the curve (iAUC) after an intravenous glucose tolerance test was not different between LDim and LD, either when the two rhythms in the LDim animals were aligned (P = 0.715) or misaligned (P = 0.368). Within the animals exposed to LDim, there was no difference in iAUC between the misaligned and the aligned state (P = 0.902) (Supplementary Figure 4).

## Discussion

In the present paper we show that in the male Wistar rat, LDim strongly reduced the daily rhythms in sleep-wake behavior. LDim acutely increased sleep in the active phase during the first dim light (active) phase with a further increase over the following days, whereas LDim gradually decreased sleep during the light (inactive) phase. The reduced sleep-wake rhythm amplitude was paralleled by reduced rhythms in the 16–19 Hz EEG frequency domain and SCN clock gene expression. In a circadian analysis, LDim surprisingly induced an endogenous free running rhythm in locomotor activity with a period of ~25 h that interacted with the entrained 24 h rhythm. The reduced daily rhythms of sleep, food intake, and energy expenditure in LDim did not cause obesity or impaired glucose tolerance.

Already during the first 12 hours of dim light exposure, LDim reduced waking and increased NREM and REM sleep. This acute increase in NREM and REM sleep may be a direct effect of the dim light on sleep regulatory areas of the brain such as the ventrolateral preoptic nucleus or the ventral subparaventricular zone, since these areas receive direct information from intrinsically photosensitive retinal ganglion cells (ipRGCs)^27^ and ipRGCs are sensitive to dim light levels^28^. Alternatively, the acute increase of NREM and REM sleep due to LDim may be mediated by decreased melatonin levels, as the dim light levels used are sufficient to decrease melatonin levels^29^ and increased melatonin has been reported to reduce REM and NREM sleep in rats^30^.

The effects of LDim on sleep increased over time. While LDim did not affect vigilance states in the light phase directly following the first 12 hours of dim light exposure, after 7 days LDim decreased NREM sleep during the light phase, and after 14 days LDim also increased waking during the light phase. The rhythm in NREM sleep slow wave activity, representing homeostatic sleep drive in the rat^31^,^32^,^33^ was nearly abolished after 2 weeks of LDim exposure, in accordance with a near-equal distribution of sleep over the 24-h cycle. LDim also strongly decreased the rhythm in the 16–19 Hz frequency domain, which is within the NREM sleep EEG frequency range identified to reflect the rhythm of the endogenous circadian timing system in the Wistar rat^33^,^34^.

Together, the gradual increase of the effect of LDim on waking, NREM sleep, and REM sleep over the days, and the loss of rhythm in the 16–19 Hz frequency domain indicated an effect of LDim on the endogenous circadian timing system. To further investigate the effect of LDim on the endogenous period and rhythm strength, we exposed animals to LDim for 30 days followed by continuous darkness. Strikingly, in all animals LDim induced a free running rhythm in locomotor activity with a period of 25.1 h that interfered with the 24-h rhythm. LDim persistently affected the endogenous rhythm, since the transfer from LDim to DD resulted in a free running rhythm with a period of 24.8 h and low rhythmic strength, whereas the transfer from LD to DD resulted in a free running rhythm with a period that was close to 24 h and a higher rhythmic strength. Theoretically, the occurrence of two interacting rhythms in locomotor behavior in LDim can be due to either two separate endogenous rhythms or one free running endogenous rhythm with a period of 25.1 h in combination with one rhythm that is synchronized to the external 24 h rhythm (masking). After transfer from LDim to DD, rhythmic strength was lower in the initial 10 days (with three animals being virtually arrhythmic), compared to the second 10 days in DD, which supports the notion that the two rhythms may be two desynchronized endogenous rhythms that gradually resynchronize over time in DD.

In a control experiment we verified whether the occurrence of two interacting free running rhythms was specific for LDim and not due to continuous exposure to minimally 5 lux. Indeed, under continuous dim light (DimDim) or continuous light (LL), no rhythm splitting was observed. Instead, in DimDim, rats showed a single free running rhythm with a period duration of 24.9 hr, which was comparable to the period duration of the free running component under LDim. The increased free running period duration in continuous dim light compared to the free running period in continuous darkness after LD is in line with Aschoff’s rule that predicts an increase of period duration with increasing intensity of light exposure in nocturnal animals^35^,^36^,^37^. The complete loss of rhythmicity that we observed in LL has been reported before under high light intensities^37^,^38^,^39^.

The interaction within one animal of two rhythms in locomotor activity with a different period, also called internal desynchronization, has been described previously in various desynchronization paradigms in the vole^40^, the mouse^41^,^42^, and the rat^43^. However, to the best of our knowledge, two interacting rhythms in locomotor activity have never been described in a 12:12 L:D lighting schedule. Thus, our present data, for the first time, indicate that internal desynchronization can occur in a situation when the light-dark cycle adheres to the natural cycle duration of 24 hr. This finding may have translational consequences, since humans usually adhere to a 24-hr cycle.

The rodent and human circadian timing system share many common features. Both in rodents and humans, the circadian effects of light are mediated by ipRGCs. In both species, these ipRGCs detect light with the photopigment melanopsin that has maximal sensitivity for blue light with a wavelength of approximately 480 nm, and integrate this information with input from the classical photoreceptors in retinal rods and cones^44^. Subsequently, ipRGCs transfer the integrated light information to hypothalamic areas including the SCN^27^,^44^. The SCN rhythm of firing activity and neuropeptide expression is largely similar between nocturnal and diurnal mammalian species^45^. Despite these similarities, there are differences between the rodent and human circadian timing system in the sensitivity to light^44^: In humans for example, light with an intensity of 1 lux is barely sufficient to entrain the circadian pacemaker^46^, whereas in rodents, approximately 10-fold lower light levels clearly affect SCN neuronal firing^47^ and circadian period^48^. In the present paper we used 5 lux as a light stimulus in order to replicate commonly used dim light conditions^18^,^24^,^25^. We are aware that lux is a measure defined by a weighting function specifically designed for the spectral sensitivity of the human image-forming visual system. Unfortunately, at present there is no validated alternative weighting function for the circadian effects of light^44^, although there have been some first attempts^49^,^50^.

The translational value of our findings is limited to the effects of dim light at night on the circadian timing system, since the direct effects of light at night is opposite between species: in rodents bright light induces sleep^16^ whereas in humans bright light induces waking^51^.

With these caveats in mind, we believe that it is important to realize that internal desynchronization due to light at night as described in the present study, might be responsible for the association between light at night and insomnia in humans. It is interesting to note that the light intensity of 5 lux is frequently encountered in human bedrooms^13^, but keeping in mind the differences in sensitivity to light between the rodents and the human circadian timing system, it will be important to investigate if these light levels also cause internal desynchronization in diurnal species.

It is well known that the rodent SCN contains multiple cell groups that can function as separate pacemakers with a different period length in terms of clock gene expression^43^,^52^ and neuropeptide secretion^53^,^54^,^55^. In Wistar rats subjected to a 22-h LD cycle, two interacting rhythms in behavior were coupled to two separate subdivisions of the SCN with the dorsomedial SCN representing the rhythm with a period of 24.9 h, and the ventrolateral SCN representing the rhythm with a period of 22 h^43^. In order to investigate the representation within the SCN of the two rhythmic components that we observed in locomotor behavior, we analyzed SCN clock gene expression at the time when the two rhythmic components in behavior were aligned or misaligned. LDim caused an overall reduction of the amplitude of the daily rhythm of *Per1* and *Arntl* expression, but no significant desynchronization between ventrolateral and dorsomedial SCN could be detected. Possibly, the sensitivity of the two time-point analysis is insufficient to detect two independent rhythms in our model. Alternatively, the spatiotemporal organization of the two presumed pacemakers within the SCN may be less pronounced in our model compared to the 22-h day paradigm^43^. Future studies with transgenic clock-gene luciferase rats^56^ may elucidate the anatomical representation of the free running rhythm.

Thus, we introduce a rat model for the effects of dim light at night on the circadian control of sleep. In contrast to the Swiss Webster mouse model^25^, we found profound disturbances of vigilance state distribution and sleep architecture. Based on the mouse model^18^,^24^, we expected that the circadian disruptions would have adverse metabolic consequences. However, despite increased daytime food intake during LDim on both chow and HFD, we did not observe effects of LDim on body weight, adiposity or glucose tolerance. On the chow diet, animals exposed to LDim did show a slightly smaller increase of food intake over the weeks compared to animals exposed to LD. Since body weight gain was not affected by the light schedule, this suggests that LDim may induce slightly more weight gain per g of food consumed (i.e. higher feeding efficiency). However, when the animals were induced to become obese with a high fat diet, this effect was not reproduced. The absence of metabolic effects contrasts to the Swiss Webster mouse model, where increased daytime food intake due to dim light at night causes obesity and decreased glucose tolerance^18^,^24^. The differences between the mouse model and our rat model may be attributable to species differences in the susceptibility to sleep disturbance or metabolic disease^57^,^58^ or to the difference in light phase duration since the Swiss Webster mouse model utilized a 16:8^18^ or 14:10^24,25^ L:Dim cycle whereas we used a 12:12 L:Dim cycle.

In conclusion, we present the first rodent model for disrupted circadian control of sleep-wake rhythms due to light at night. In male Wistar rats, LDim strongly reduced the daily amplitude of sleep-wake and feeding-fasting behavior. This amplitude reduction was due to a direct sleep promoting effect combined with reduced SCN output strength due to internal desynchronization between the environmentally entrained 24 h rhythm and the LDim induced free running rhythm of ~25 h. We show that internal desynchronization is possible in a 12:12 L:D cycle, and therefore identified a potential mechanism for the association between light at night and insomnia in humans. Taken together, the detrimental effects of artificial light at night on the circadian control of sleep warrant attention from policy makers, researchers, and anyone with the control over a light switch.

## Methods

### Animals

Male Wistar WU rats (Charles River, Germany) were used for all experiments. Before the start of experimental procedures rats were given at least one week time for accommodation to the animal facility with standard chow and water available *ad libitum*. Experimental protocols were approved by the Animal Experiments Ethical Committees of the Leiden University Medical Center and/or the Royal Netherlands Academy of Arts and Sciences, and the study was performed in accordance with Dutch laws.

### Light schedules

During accommodation, rats were exposed to a 12-h light (150–200lux):12-h dark (LD) schedule. To investigate the effect of dim light at night, rats were exposed to a 12 h light (150–200 lux):12 h dim white light (5 lux) (LDim) schedule, while control animals continued exposure to the LD schedule. During vigilance state and circadian activity experiments, dim light was emitted by white fluorescent tubes placed above the cage. During SCN clock gene expression and metabolic experiments, dim light was emitted by white LED lights placed around the cage. All light intensities were verified in the animal gaze direction with an AvaSpec 2048-SPU (Avantus BV, the Netherlands) light meter. The light source spectral power distributions are shown in Supplementary Figure 5.

### Vigilance state experiment

Rats (n = 13; bodyweight 283 ± 5 g) were anesthetized with Ketamine/Xylazine/Atropine, fixed in a stereotaxic frame and implanted with 2 electroencephalogram (EEG) screw electrodes (Plastics One, Roanoke, VA, USA; 1.4 mm diameter) and 2 electromyogram (EMG) patch electrodes (Plastics One; 0.35 mm diameter) as described previously^26^,^31^,^32^,^33^. One EEG electrode was placed over the right hemisphere above the sensory cortex, while the other was placed above the cerebellum. The EMG electrodes were inserted between the neck muscles and the skin. The wires of the electrodes were set in a plastic pedestal (Plastics One) which was fixed to the skull with dental cement and 3 additional support screws.

After 7–10 days recovery from surgery in individual housing, rats were divided into two bodyweight-matched groups and exposed to either LDim (n = 8) or LD (n = 5). Twenty-four h EEG/EMG recordings were performed at baseline (day 0, prior to the LDim schedule), day 1, day 7 and day 14. Recordings on day 0 and day 1 started at ZT12, recordings on day 7 and day 14 started at ZT0. The EEG and EMG were recorded with a portable system (Institute of Pharmacology and Toxicology, University of Zurich, Zurich, Switzerland) as described previously^59^,^60^. Before each recording, a calibration signal (10 Hz sine wave, 300 μV peak-to-peak) was recorded on the EEG and EMG channel. Both signals were amplified (amplification factor ~2000), conditioned by analogue filters (high-pass filter −3 dB at 0.16 Hz) and sampled at 512 Hz. The signals were filtered through a digital Finite Impulse Response filter: EEG low-pass filter at 30 Hz and EMG band-pass filter between 20 and 40 Hz.

The vigilance states (waking, NREM sleep and REM sleep) were scored off-line in 4-s epochs by visual inspection of the raw EEG and EMG signal, as well as EEG power density in the slow-wave range (0.75–4.0 Hz) according to standardized criteria for rats^26^,^61^. Artifact free EEG power density values were computed for 4-s epochs in the range between 0.25–25.0 Hz^59^,^60^,^62^. Between 0.25 and 5.0 Hz, values were collapsed into 0.5 Hz bins, between 5.25–25.0 Hz in 1-Hz bins. Average values of the amount of each vigilance state and the corresponding spectra, and changes over the day in vigilance states and EEG power density, were computed for each vigilance state and for each recording day. EEG power density data were standardized relative to the individual 24-h mean baseline value in NREM sleep (=100%) and subsequently averaged over all animals.

### Circadian experiments

Rats (n = 8, bodyweight 254 ± 1 g) were individually housed in transparent plastic cages and locomotor activity was monitored by passive infrared detectors using Actimetrics software (Wilmette, Il, USA). In circadian experiment A, animals were first exposed to 10 days LD and subsequently to 30 days LDim, 20 days constant darkness (DD), re-entrainment during 30 days LD and finally 10 days DD. In circadian experiment B we investigated the effect of continuous light intensity on circadian activity by exposing the rats (n = 8, bodyweight 251 ± 2 g) consecutively to at least 10 days LD, 16 days constant dim light (DimDim), 22 days constant light (LL, 150–200 lux), 32 days DimDim, 14 days LD and 16 days DimDim.

### SCN clock gene expression experiment

Individually housed rats (n = 42, bodyweight 254 ± 4 g) were exposed to either LDim or LD and locomotor activity was measured using pressure-sensitive baseplates^5^. For LDim animals, the moments were determined when the activity phase of the free running rhythm and the activity phase of the 24-h rhythm were either aligned or misaligned, as determined by visual inspection of the actogram combined with F-periodogram analysis^63^,^64^. Rats were divided into 6 groups (n = 7 per group) and sacrificed accordingly: 1) LDim aligned ZT6, 2) LDim aligned ZT18, 3) LDim misaligned ZT6, 4) LDim misaligned ZT18, 5) LD ZT6 and 6) LD ZT18. Animals were briefly anesthetized with 80% CO_2_ and decapitated. Brains were frozen on dry ice and stored at −80 °C. Coronal 20 μm slices of the SCN were obtained with a cryostat. *In situ* hybridization of *Per1* and *Arntl (Bmal1*) were performed using riboprobes of r*Per1* (kindly provided by Dr. H. Okamura, Kyoto University, Japan) and m*Arntl* (m*Bmal1*)^65^ genes. Antisense RNA probes were generated with an *in vitro* transcription kit (Maxiscript; Ambion, Austin, TX, USA). Hybridization was carried out as described previously^66^. Slices and radioactive standards were exposed to an autoradiographic film (Biomax MS-1 Kodak, Sigma-Aldrich, St Louis, MO, USA). Quantitative analysis of the autoradiograms was performed using the ImageJ software (W. Rasband, National Institutes of Health, Bethesda, MD, USA). A Nissl staining was performed on the same slides and the section containing the mid SCN was selected. The optical density of the whole SCN, the ventromedial SCN and the dorsolateral SCN were measured by a researcher (JMen) who was blind for treatment, Relative optical density was determined by subtracting the background intensity (measured in the anterior hypothalamic area) from the signal for each animal. Left and right sided relative optical densities were averaged and the dorsomedial/ventrolateral ratio^43^ was calculated.

### Metabolic experiments

The first metabolic experiment was performed with 8 rats (bodyweight 201 ± 2 g) on a regular chow diet (Harlan Teklad Global Diet 2918, Harlan Laboratories Inc, Madison, Wisconsin, USA). Individually housed rats were divided into two bodyweight-matched groups and exposed to either LDim or LD. For the assessment of food intake and energy expenditure, rats were placed in PhenoMaster/LabMaster calorimetric cages (TSE Systems GmbH, Bad Homburg, Germany) in week 0 (baseline, before start of dim light exposure), and 1, 2 and 7 weeks after the start of dim light exposure. Rats were sacrificed after 8 weeks. The second metabolic experiment was performed with 16 rats (bodyweight 196 ± 1 g) on a high fat diet (HFD) (D12451 high fat diet, Research Diets, Inc, New Brunswick, New Jersey, USA). Again, rats were divided into two bodyweight-matched groups and exposed to either LDim or LD. HFD animals were placed in the calorimetric cages in week 0 (baseline, before start of dim light exposure), and 1, 2, 3, 4, 5, 8 and 9 weeks after the start of dim light exposure, and HFD animals were sacrificed after 11 weeks. Rats were given at least 2 hours to accommodate to the calorimetric cages before the 48-hr recordings started.

After sacrificing the animals, the mesenteric, epidydimal, perirenal and subcutaneous WAT depots, adrenals and thymus from the right side of the rat were collected and weighed.

### Glucose tolerance experiment

Individually housed rats were divided into two bodyweight-matched groups (n = 7 per group) and exposed to either LDim or LD for at least 7 days before surgery. Locomotor activity was measured using pressure-sensitive baseplates^5^. The rats (bodyweight 320 ± 11 g at surgery) were anesthetized with fentanyl/fluanisone/midazolam, a silicone catheter was inserted into the right atrium via the jugular vein and attached to the skull with 4 screws and dental cement^5^. Rats were given at least 1 week to recover, and glucose tolerance was assessed at ZT6 twice in every rat: at the time when the activity phases of the free running and 24 h rhythms were aligned and when the activity phases were misaligned (as determined by visual inspection of the actogram combined with F-periodogram analysis^63^,^64^). One day before the intravenous glucose tolerance test (IVGTT)^5^ rats were attached to a head stage that allows infusion and blood sampling in freely moving animals. On the day of the experiment, food was removed 4 h before the glucose injection. A 1000 mg/kg bodyweight glucose bolus (Sigma-Aldrich) was injected at t = 0 and blood samples were obtained from the catheter at t = −1, 5, 10, 20, 30 and 60 min. Glucose was measured immediately with a FreeStyle Freedom Lite glucose measurement device (Abott Diabetes Care, Alameda, CA, USA).

### Statistical analysis

Normally distributed variables are expressed as mean and standard error of the mean (SEM). Vigilance state and EEG data were analyzed with repeated measures ANOVA with the factors *Phase* (Light, Dark/Dim), *Day* (0, 1, 7, 14) and their *Interaction.* Power density spectra of NREM sleep were analyzed with ANOVA with the factors *Frequency* (0.25–25.0 Hz), Day (0, 1, 7, 14) and their *Interaction*. For circadian locomotor activity data, the rhythm period and strength were determined with F-periodogram analysis^63^ as previously described^64^. Rhythm strengths were analyzed by ANOVA with the factor *Light schedule* (LD, LDim, DD). SCN gene expression data were analyzed with ANOVA using the factors *Time* (ZT6, ZT18), *Group* (LD, LDim-aligned, LDim-misaligned) and their *Interaction*.

Data from the calorimetric cages were analyzed with a linear mixed model in order to correctly incorporate missing data, with *Light schedule* (LD, LDim), *Week* (week 0–9) and their *Interaction* as fixed effects. The covariance structure ‘Compound Symmetry’ was selected based on the Akaike’s Information Criterion (AIC).

If a significant main effect was detected in ANOVA or linear mixed model analyses, post hoc tests were performed with an independent samples two-tailed student’s *t*-test (for 2 groups with independent samples), a related samples two-tailed student’s *t*-test (for 2 groups with related samples) or Duncan’s test (for >2 groups).

For glucose tolerance data, the iAUC was calculated for individual animals as described previously^67^. The effect of LDim exposure on body weight, organ weight and glucose tolerance was assessed with a student’s independent samples two-tailed *t*-test or a student’s related samples two-tailed *t*-test where appropriate.

All statistical analysis were performed with SPSS statistics (v21, SPSS, Inc) using a significance level of 0.05.

## Additional Information

**How to cite this article**: Stenvers, D. J. *et al*. Dim light at night disturbs the daily sleep-wake cycle in the rat. *Sci. Rep.* **6**, 35662; doi: 10.1038/srep35662 (2016).

## Supplementary Material

Supplementary Information

## Figures and Tables

**Table 1:** LDim induces an endogenous free run interacting with the entrained 24 hr rhythm in locomotor activity.

Circadian experiment A (n = 8)			Circadian experiment B (n = 8)		
	Period	Strength		Period	Strength
**LD** (10 days)	24.0 ± 0.0^A^	0.38 ± 0.02^A^	**LD** (10 days)	24.0 ± 0.0^A^	0.21 ± 0.00^A^
**LDim** (30 days)	1) 23.9 ± 0.0^A^ 2) 25.1 ± 0.0^B^	1) 0.17 ± 0.01^B^ 2) 0.09 ± 0.01^C^	**DimDim** (16 days)	24.9 ± 0.3^B^	0.11 ± 0.00^B^
**DD1** (10 days)	24.8 ± 0.3^B^	0.11 ± 0.02^C^	**LL** (22 days)	Arrhythmic	n/a
**DD2** (10 days)	24.5 ± 0.1^AB^	0.18 ± 0.02^B^	**DimDim** (32 days)	Arrhythmic	n/a
**LD** (30 days)	24.0 ± 0.0^A^	0.21 ± 0.02^AB^	**LD** (14 days)	24.0 ± 0.0^A^	0.20 ± 0.02^A^
**DD** (10 days)	24.2 ± 0.1^A^	0.14 ± 0.01^B^	**DimDim** (16 days)	24.8 ± 0.4^B^	0.06 ± 0.01^C^

Within each experiment values that are annotated by different letters are significantly different from each other (Duncan P < 0.05, after a significant main effect of *Light* in the ANOVA analysis). Data are represented as mean ± SEM. DD, continuous darkness. DimDim, continuous dim white light. LL continuous light. n/a, not applicable.
