# Supplementary material for: Dim light at night disturbs the daily sleep-wake cycle in the rat
Source: Sci Rep. 2016 Oct 20;6:35662. doi: 10.1038/srep35662 (PMC5071835; doi:10.1038/srep35662)
Supplement: Supplementary Information [file srep35662-s1.pdf]

SUPPLEMENTARY INFORMATION

Dim light at night disturbs the daily sleep-wake cycle in the rat

Dirk Jan Stenvers MD[1*], Rick van Dorp BSc[1], Ewout Foppen BSc[1], Jorge Mendoza PhD[2], Anne-Loes Opperhuizen MSc[3], Eric Fliers MD PhD [1], Peter H. Bisschop MD PhD[1], Johanna H Meijer PhD [4], Andries Kalsbeek PhD [1,3], Tom Deboer PhD[4]

[1] Department of Endocrinology and Metabolism, Academic Medical Center (AMC), University of Amsterdam, Amsterdam, The Netherlands

[2] Institute of Cellular and Integrative Neurosciences, CNRS UPR3212, University of Strasbourg, France

[3]Hypothalamic Integration Mechanisms, Netherlands Institute for Neuroscience (NIN), Royal Netherlands Academy of Arts and Sciences, Amsterdam, The Netherlands

[4]Laboratory for Neurophysiology, Department of Molecular Cell Biology, Leiden University Medical Center, Leiden, The Netherlands

Supplementary material: 5 supplementary figures, 2 supplementary tables

Supplementary Table 1. LDim induces a slight decrease in total 24-h food intake with a chow diet, but not with a high fat diet.

week	Food intake (kJ/24h)				Energy expenditure (kJ/24h)			
	Chow (n=8)[A]		HFD (n=16)[B]		Chow (n=8)[C]		HFD (n=16)[D]	
	LD	LDim	LD	LDim	LD	LDim	LD	LDim
0	273 ± 5	269 ± 5	356 ± 6	329 ± 11	212 ± 4	215 ± 5	212 ± 3	212 ± 5
1	284 ± 9	281 ± 4	340 ± 8	312 ± 10	245 ± 8	237 ± 3	225 ± 3	216 ± 4
2	301 ± 9	287 ± 3	338 ± 18	340 ± 14	260 ± 10	246 ± 6	252 ± 11	247 ± 11
3			368 ± 12	335 ± 14			221 ± 20	232 ± 9
4			347 ± 10	345 ± 12			261 ± 11	254 ± 16
5			340 ± 10	340 ± 12			254 ± 7	246 ± 10
7	312 ± 11	281 ±13			294 ± 4	271 ± 7		
8			348 ± 11	319 ± 12			273 ± 6	259 ± 5
9			315 ± 11	313 ± 3			271 ± 3	255 ± 5

[A]On a chow diet, LDim induced a small decrease of total 24-h food intake compared to LD (linear mixed model, *Light schedule* P=0.927, *Week* P=0.002, *Interaction* P=0.025). Post hoc independent samples student's *t*-tests did not detect significant differences between LD and LDim for individual weeks.

[B]On a high fat diet (HFD), total 24-h food intake was not affected by LDim (linear mixed model, *Light schedule* P=0.079, *Week* P=0.321, *Interaction* P=0.238)

[C]On a chow diet, total 24-h energy expenditure was not affected by LDim (linear mixed model, *Light schedule* P= 0.908, *Week* P<0.001, *Interaction* P=0.084).

[D]On a HFD, total 24-h energy expenditure was not affected by LDim (linear mixed model, *Light schedule* P=0.990, *Week* P<0.001, *Interaction* P=0.249).

Supplementary Table 2. LDim does not affect body weight or adiposity.

	Chow (n=8)			HFD (n=16)		
	LD	LDim	P value[A]	LD	LDim	P value[A]
Mesenteric WAT(mg)	5.0 ± 0.2	4.4 ± 0.3	0.095	12.1 ± 0.6	12.4 ± 1.0	0.775
Epidydimal WAT(mg)	2.8 ± 0.1	2.7 ± 0.2	0.728	7.0 ± 0.2	6.9 ± 0.5	0.822
Perirenal WAT(mg)	3.2 ± 0.5	3.3 ± 0.3	0.882	10.0 ± 0.4	11.0 ± 0.9	0.306
Subcutaneous WAT(mg)	2.6 ± 0.1	2.4 ± 0.1	0.268	5.5 ± 0.4	6.0 ± 0.6	0.514
Adrenals (ug)	46 ± 1	43 ± 2	0.271	35 ± 6	38 ± 5	0.736
Thymus (mg)	440 ± 16	466 ± 16	0.286	480 ± 12	513 ± 17	0.130
Total WAT(mg)	13.6 ± 0.6	12.8 ± 0.8	0.424	34.7 ± 1.4	36.4 ± 2.9	0.601
Bodyweight (mg)	412 ± 6	403 ± 9	0.380	510 ± 6	523 ± 14	0.375

[A]Differences between LD and LDim were determined with a student's independent samples t-test.

Data are expressed as mean ± SEM

HFD, high fat diet. WAT, white adipose tissue.

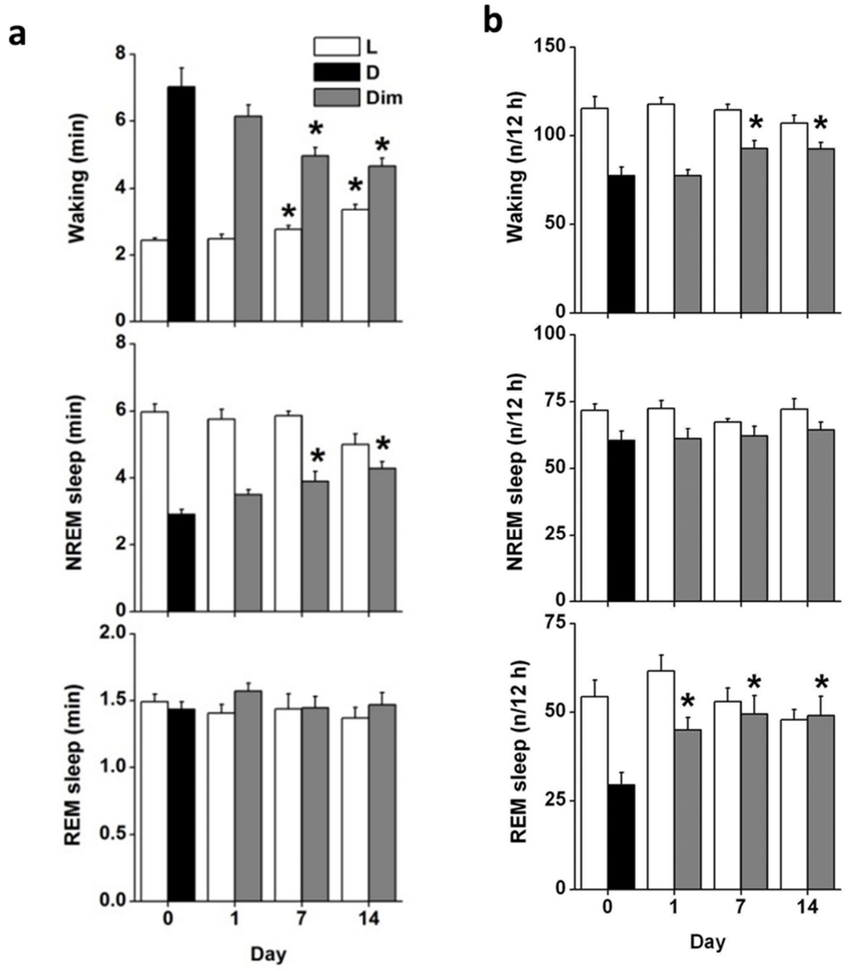

Supplementary Figure 1. LDim reduces the daily rhythm of waking and NREM sleep episode durations as well as waking and REM sleep episode numbers. a. Duration of episodes of waking (top), NREM sleep (middle) and REM sleep (bottom) during the light (open bars), dark (black bars) and dim light (grey bars) phase. For waking, LDim exposure induced a reduction of episode duration in the dark phase and an increase of episode duration in the light phase (*Phase* $P<0.001$, *Day* $P=0.009$, *Interaction* $P<0.001$). For NREM sleep, LDim exposure caused an increase of episode duration during the dark phase (*Phase* $P<0.001$, *Day* $P=0.248$, *Interaction* $P<0.001$). REM sleep episode duration was not affected (*Phase* $P=0.386$, *Day* $P=0.767$, *Interaction* $P=0.523$). **b.** Number of episodes of waking (top) NREM sleep (middle) and REM sleep (bottom) during the light (open bars), dark (black bars) and dim light (grey bars) phase. LDim exposure caused a slight increase in the number of waking episodes in the dark phase (*Phase* $P<0.001$, *Day* $P=0.401$, *Interaction* $P=0.014$) and an increase in the number of REM sleep episodes in the dark phase (*Phase* $P<0.001$, *Day* $P=0.025$, *Interaction* $P=0.005$). The number of NREM sleep episodes was not affected (*Phase* $P<0.001$, *Day* $P=0.746$, *Interaction* $P=0.757$).

Significant ($P<0.05$) differences from the baseline measurement are indicated by asterisks. Data are means ± SEM; n=8.

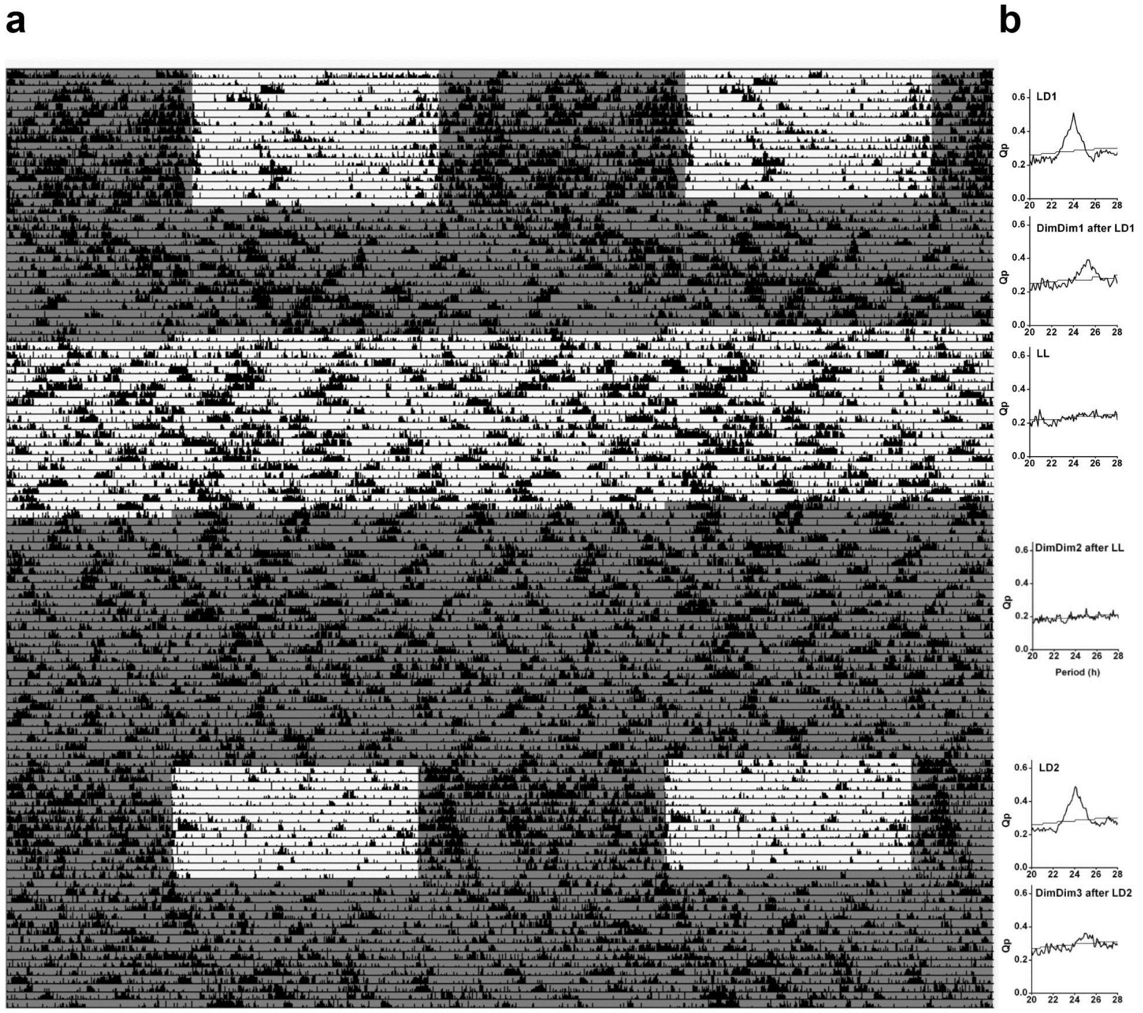

Supplementary Figure 2. Exemplar actogram and periodogram of circadian experiment B. We exposed Male Wistar rats consecutively to at least 10 days LD, 16 days constant dim light (DimDim), 22 days constant light (LL), 32 days DimDim, 14 days LD and 16 days DimDim. In none of the DimDim periods, dual rhythms could be detected. In LL and the second period in DimDim (after LL), the animals were arrhythmic.

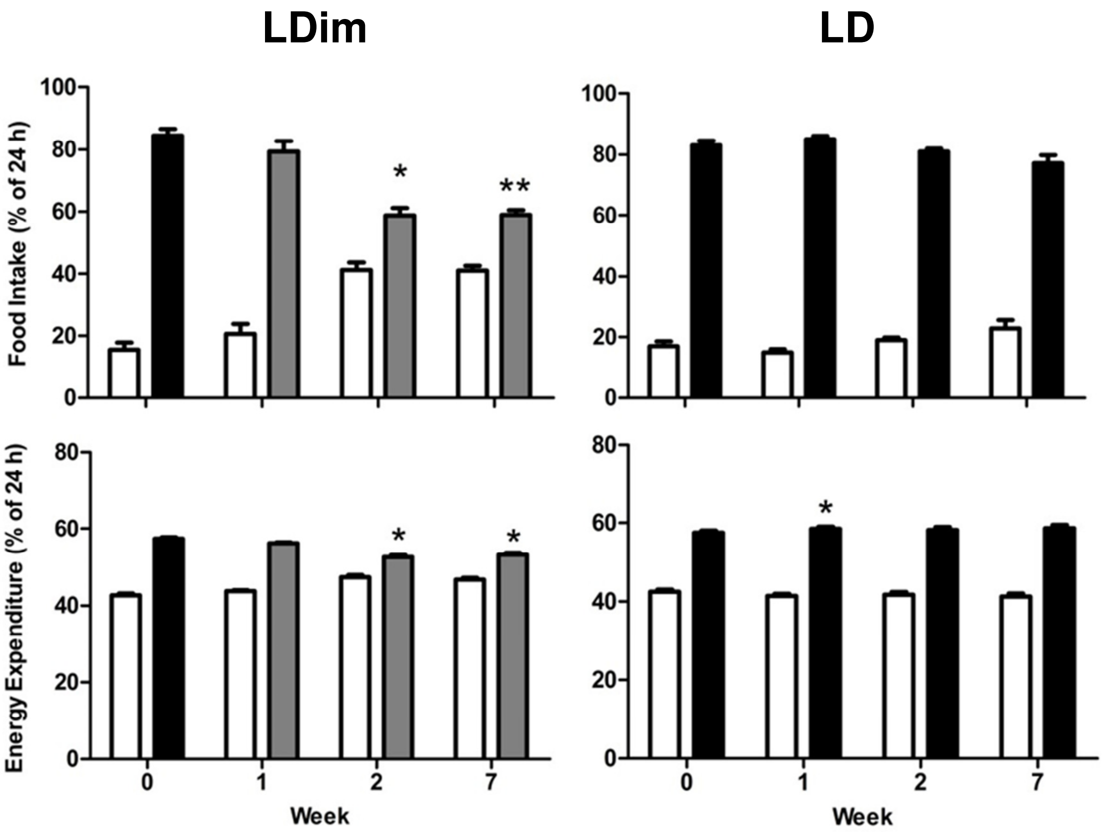

Supplementary Figure 3. **LDim reduces the daily rhythm of food intake and energy expenditure on a chow diet.** On a chow diet, LDim exposure (left) induced a decreased amplitude of the daily rhythm in food intake and energy expenditure compared to LD animals (right). Significant differences from baseline are indicated by asterisks ($P<0.05$) or double asterisks ($P<0.001$). Data are means ± SEM; n=8.

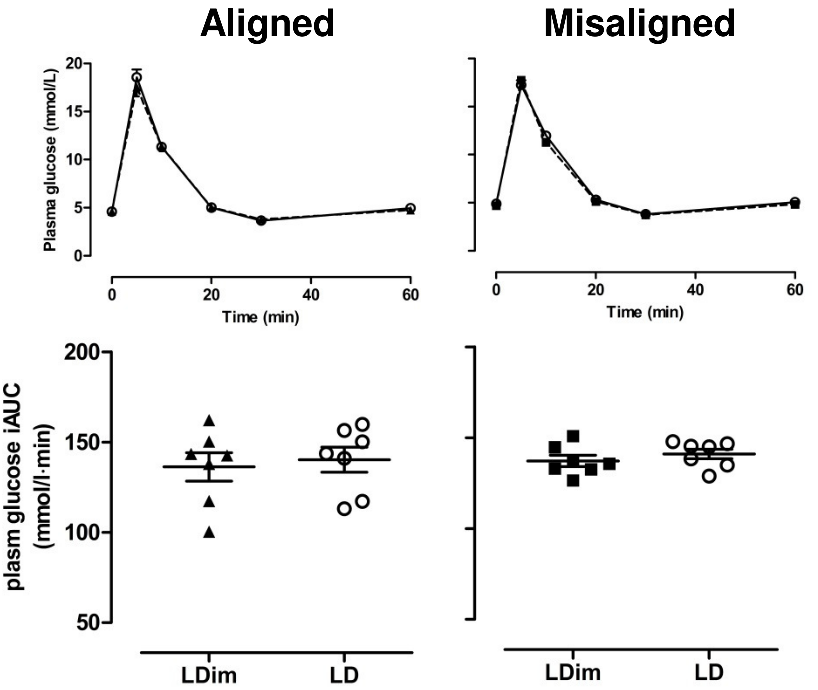

Supplementary Figure 4. LDim has no effect on glucose tolerance. Rats were fed a chow diet and either exposed to LD (open circles) or LDim. Glucose tolerance was assessed by an intravenous glucose tolerance test when the free running rhythm and the entrained 24-h rhythm in the LDim group were aligned (closed triangles) or misaligned (closed squares). Bottom figures: individual iAUC values.

Data are means ± SEM; n=14.

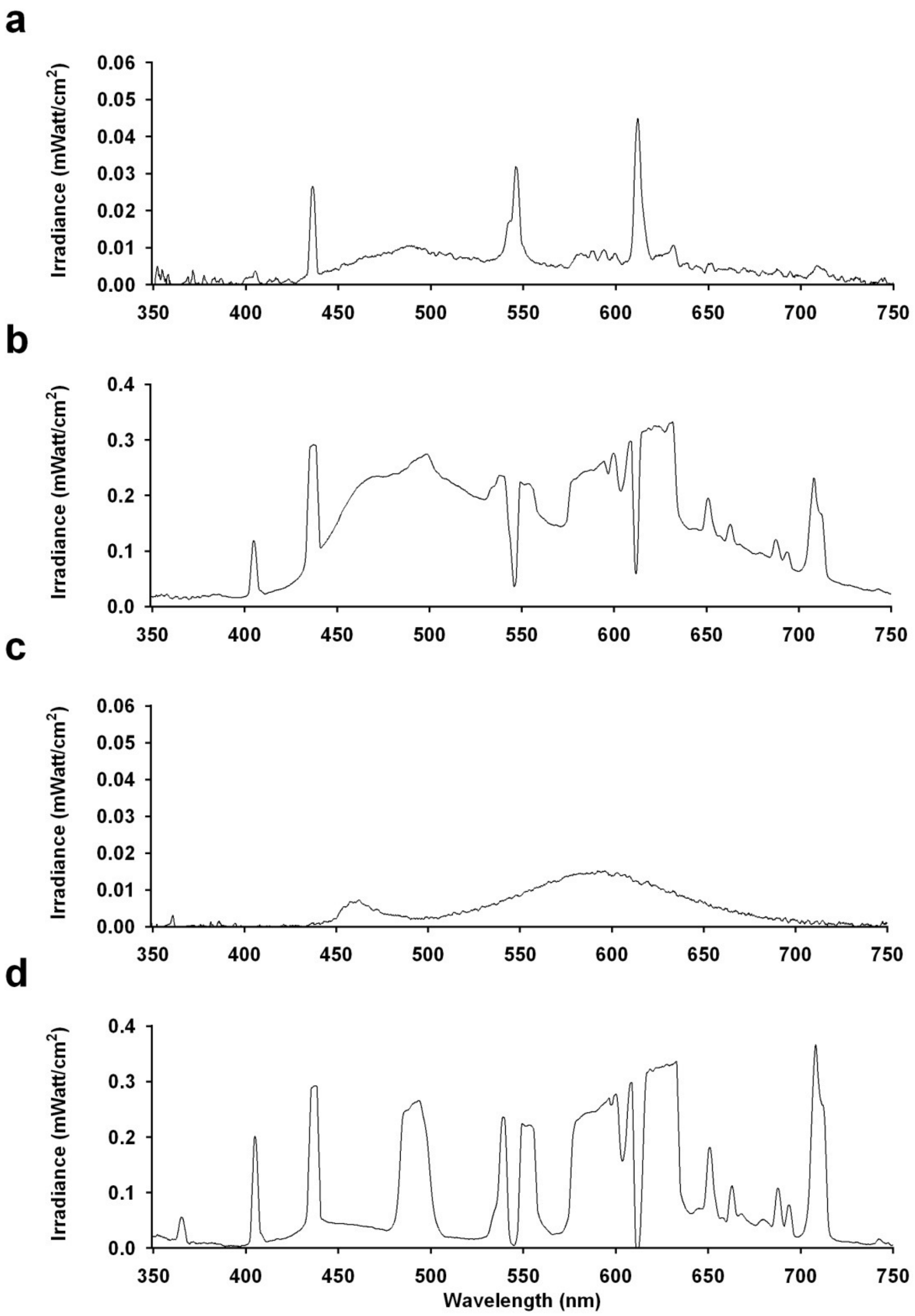

Supplementary Figure 5. Spectral power distributions of light sources. a. Dim light (5 lux) and **b.** bright light (150-200 lux) emitted by white fluorescent tubes for vigilance state and circadian activity experiments. **c.** Dim light (5 lux) emitted by white LED lights and **d.** bright light (150-200 lux) emitted by white fluorescent tubes for SCN clock gene and metabolic experiments. Note the 10 fold difference in y-axis units between dim and bright light. Spectral power distributions were measured with an AvaSpec 2048-SPU (Avantus BV, the Netherlands) light meter.
